# Analyzing body dissatisfaction and gender dysphoria in the context of minority stress among transgender adolescents

**DOI:** 10.1186/s13034-024-00718-y

**Published:** 2024-03-02

**Authors:** Alexandra Brecht, Sascha Bos, Laura Ries, Kerstin Hübner, Pia-Marie Widenka, Sibylle Maria Winter, Claudia Calvano

**Affiliations:** 1 Department of Education and Psychology, Clinical Child and Adolescent Psychology and Psychotherapy, Freie Universität Berlin, 14195 Berlin, Germany; 2 Department of Child and Adolescent Psychiatry, Charité-Universitätsmedizin Berlin, corporate member of Freie Universität Berlin, Humboldt Universität zu Berlin and Berlin Insitute of Health, 13353 Berlin, Germany; 3 Klinik für Kinder- und Jugendmedizin, Universität zu Lübeck, Ratzeburger Allee 160, 23562 Lübeck, Germany

**Keywords:** Transgender adolescents, Body dissatisfaction, Gender dysphoria, Minority stress, Poor peer relations, Trans hostility

## Abstract

**Background:**

Gender dysphoria among transgender adolescents has predominantly been examined in relation to body dissatisfaction. While in adult transgender samples, body dissatisfaction is higher than in cisgender controls, this has so far rarely been investigated for adolescents. In the context of a cisnormative society, the impact of influences from the social environment on body dissatisfaction and gender dysphoria has been neglected in research. Therefore, this study aimed to (1) provide a detailed analysis of body dissatisfaction among young transgender people and (2) investigate whether body dissatisfaction and gender dysphoria are associated with experiences of minority stress such as trans hostility and poor peer relations (PPR).

**Methods:**

The paper presents a cross-sectional study among a sample of transgender adolescents, presenting at a specialized outpatient counseling clinic (*N* = 99; age *M* = 15.36, *SD* = 1.85). First, body dissatisfaction (assessed by the Body-Image-Scale; BIS), was explored and compared to data from a population-based control group of cisgender peers (*N* = 527; age *M* = 14.43, *SD* = 0.97). Second, within a clinic-referred transgender subsample (*n* = 74), associations between body dissatisfaction and gender dysphoria (measured by Utrecht Gender Dysphoria Scale; UGDS), PPR (measured by the Youth-Self-Report; YSR-R), and trans hostile experiences (assessed in clinical interview) were examined by correlations, t-tests and multivariate regression.

**Results:**

Transgender adolescents reported more body dissatisfaction than cisgender peers. The dissatisfaction with sex characteristics, non-hormonal reactive body regions and the total score for body dissatisfaction were positively related with gender dysphoria. The majority had experienced trans hostility in the present and/or past (54.1%) and PPR (63.5%). More body dissatisfaction was correlated with more PPR regarding visible body parts i.e., hair, overall appearance and muscles, whilst PPR and gender dysphoria were not associated. Transgender adolescents who experienced trans hostility showed higher gender dysphoria and PPR, but not more body dissatisfaction. In multiple regression, trans hostility predicted gender dysphoria, whilst age and PPR predicted body dissatisfaction.

**Discussion:**

Experiences of minority-stress differentially interact with body dissatisfaction and gender dysphoria among transgender adolescents. Social correlates of body dissatisfaction and gender dysphoria must be considered when working with young transgender people.

**Supplementary Information:**

The online version contains supplementary material available at 10.1186/s13034-024-00718-y.

## Background

Persons who do not identify with their assigned sex at birth (ASAB) identify themselves for example as trans, non-binary or gender diverse, which can be subsumed under the umbrella term *transgender*. In the ICD-11, the wide variety of not identifying with the ASAB is described as *gender incongruence*. In a population-based study of *N* = 135.760 adolescents under 21 years, 0.6% identify with the opposite sex and 3.3% as non-binary [1]. Non-binary means that a person does not identify with the gender binary, whilst trans girl refers to people assigned male at birth (AMAB) but identify as girl; trans boy describes people assigned female at birth (AFAB) but identify as boy. The term cisgender is used when a person identifies with the ASAB. In public discourse, being transgender is often accompanied by the ascription of “being trapped in the wrong body” [2]. In this context, the DSM-5 defines *gender dysphoria* as a person’s experience of desiring the bodily characteristics of another gender, in particular the primary and secondary sex characteristics, as well as the deep belief that the individual is another gender than their ASAB [3]. Thus, according to this definition, the dissatisfaction with one’s body is central to the experience of gender dysphoria. Consequently, research often focuses on the assessment of body dissatisfaction in the context of transgender person’s well-being though notably, not all transgender people experience discomfort with their body [4]. Population-based and clinical studies have consistently shown that transgender adults, compared to cisgender individuals, reported more insecurities and dissatisfaction with their own bodies and their own attractiveness and showed lower self-confidence [5–9]. However, comparable analyses for adolescents are lacking so far, although it is important to better understand transgender experiences during the critical time of puberty and to which extent the experiences differ from their cisgender peer’s. While in cisgender adolescent samples, girls consistently report higher body dissatisfaction than boys, gender differences among transgender persons are less consistent [6, 9, 10]. There have been different approaches to assess body dissatisfaction in transgender adolescents such as looking at dissatisfaction with overall appearance and weight loss [6] or the global body image [11, 12]. Besides the global level of body dissatisfaction, it might be relevant to gain insights in the experience of body dissatisfaction with specific body regions, e.g., to sexual characteristics or hormonally reactive parts, especially in a phase of puberty where significant physical changes evolve. Up to now, specific analyses of body dissatisfaction in different body regions with different functions and different levels of hormonal reactivity among transgender adolescents and a comparison of these facets of body dissatisfaction with cisgender peers are not available yet.

Undoubtedly, transgender people still represent a marginalized group in mostly cis normative (social norms that strongly rely on cisgender and binary ideas) societies and experience discrimination and (micro)-aggression on a daily basis, covered by the term *minority stress* [13]. Transgender adolescents experience bullying and poor peer relations **(**PPR**)** significantly more often than their cisgender peers [14–18]. Testa et al. [19] adapted Meyer’s *minority stress model* for transgender people stating that the chronic exposure of discrimination and hostility leads to their internalization (*Internalized Transphobia*) and eventually auto-aggression, self-depreciation and other related mental health issues. These assumptions are supported by various studies [20–22].

Therefore, when addressing gender dysphoria among transgender persons, we need to consider experiences of discrimination and trans hostility as crucial social influences. Studies with transgender adults found that discrimination and harassment had an adverse impact on transgender individual’s body appreciation [2], and that minority stress explained both disordered eating and body dissatisfaction [23, 24]. An online survey [25] involving 923 young transgender people (aged 14–25) across Canada found that rates of harassment and discrimination were linked to higher odds of disordered eating in the past year, defined as binge eating, fasting or vomiting to lose weight, while protective factors, including family and school connectedness, caring friends, and social support were linked to lower odds of disordered eating in the last 12 months. Up to now, little is known on the impact of minority stress and a (trans hostile) social environment on gender dysphoria and body dissatisfaction among transgender adolescents.

### Aims of this study

This study first aims to analyze body dissatisfaction among clinic-referred transgender adolescents. Body dissatisfaction, both globally and with respect to specific body regions (e.g., sex characteristics, non-hormonal reactive body parts), will be explored among the transgender group (TG) and scores will be compared with reference scores derived from a large cisgender school-based sample (control group; CG). We hypothesize that body dissatisfaction, both the total score and subscale scores covering specific body regions, will be higher among the TG compared to the school-based CG. Exploratory analyses of gender identity differences will be conducted in both samples. Second, among the TG, associations between body dissatisfaction, gender dysphoria and experiences of minority stress, specifically PPR and trans hostile experiences, will be explored. We hypothesized that body dissatisfaction and gender dysphoria correlate positively with each other as well as with PPR and that body dissatisfaction, gender dysphoria and PPR will be higher in transgender adolescents reporting trans hostile experiences than in those who do not report those experiences. Third, by multiple regression, we will investigate the explanatory power of PPR and trans hostile experiences for the outcome of body dissatisfaction and gender dysphoria, controlled by age and gender identity.

## Methods

### Design and setting

Data collection for the transgender sample took place between December 2018 and September 2022 in the interdisciplinary special consultation for questions of gender identity in childhood and adolescence (German abbreviation GIF), affiliated to the Social Pediatric Center of Child and Adolescent Psychiatry at the Charité – Universitätsmedizin Berlin. The GIF is specialized in the affirmative care of children and adolescents with gender incongruence, with high relevance given to the inclusion of caregivers as well as the recognition of the individual development stage of each person. The interdisciplinary team consists of clinical psychologists, endocrinologists, psychiatrists, pediatrics and phoniatricians who provide education and treatment services, from initial psychological counseling to endocrinological and phoniatric treatment. The GIF conducts concomitant research, covering interview and questionnaire measures on gender identity and mental health at each visit. All patients included in the study were treatment naïve. Further, in every initial consultation, suicidality, self-harm, other psychological symptoms or problems and bullying experiences are explored by licensed clinical child and adolescent psychotherapists and psychiatrists. In the initial assessment, the Body Image Scale (BIS) [26], the Youth Self-Report [27] and the Utrecht-Gender-Dysphoria-Scale (UGDS) [28] are completed as standard assessments. The concomitant research is supported by a social psychiatric outpatient-clinic which offers psychotherapy weekly - if needed - for young trans and non-binary people and accompanies them before and during hormone treatment. The clinic uses the same set of questionnaires and diagnostic procedures since most adolescents come with a quest for medical affirmative treatment. The outpatient clinic also provides consultation about medical transition steps and collaborates with the respective institutions such as endocrinologists. Informed consent on concomitant research is obtained from each adolescent and caregiver.

The primary inclusion criteria for this study were the first presentation at the GIF at the Charité or the social psychiatric outpatient clinic in Berlin between December 2018 and November 2022; age between 11 and 18 years; the assignment of the diagnosis F64.0 (Transsexualism) according to ICD-10, the currently used classification system, as the ICD-11 is not yet implemented in German clinical practice; and available BIS data [26]. The age criterion was chosen based on the patient’s physical developmental state (pubertal and post pubertal) and based on the age span of the validated measures. e.g., the YSR-R.

The control data for this paper were derived from the evaluation study in the Disorder of Sex Development/Intersexuality Network, funded by the German Federal Ministry of Education and Research [30]. For this paper, the CG comprised N_CG = 527 persons, aged *M* = 14.43 years (*SD* = 0.97, range 13–16 years). 296 (56.2%) reported a female gender and 231 (43.8%) a male gender [29]. More detailed information about the evaluation study can be retrieved from Appendix 1.

### Sample characteristics

**Fig. 1:**
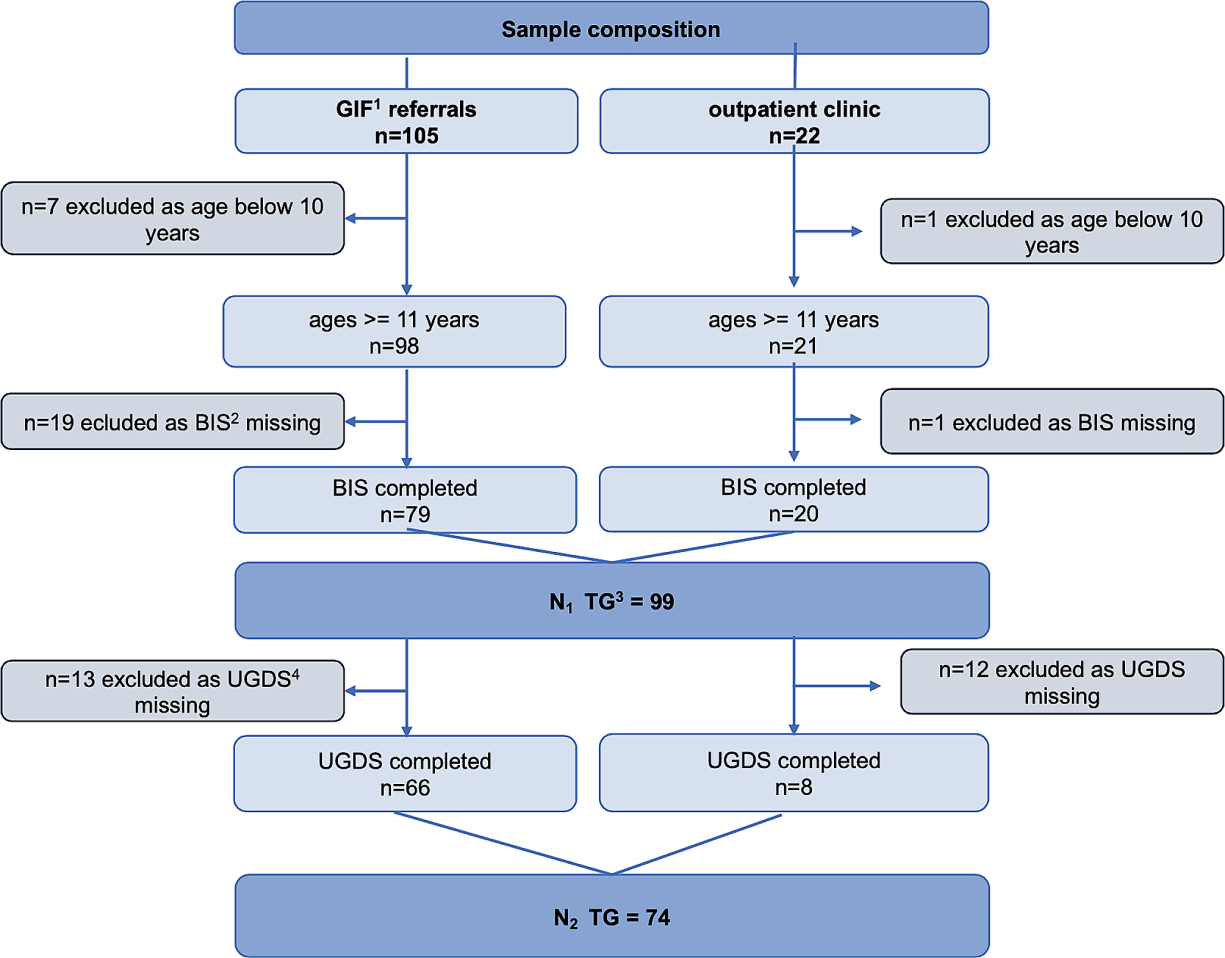
Participant flow for this study. *Notes*. ^1^ Gender Identity Service; ^2^ Body Image Scale; ^3^ transgender group; ^4^ Utrecht Gender Dysphoria Scale

A participant flow of the current study is depicted in Fig. 1. The initial sample of the clinic-referred transgender group (TG) consisted of *N* = 127 children and adolescents who consulted the two transgender outpatient clinics. For the comparison analyses with the cis gendered control group, 79 patients of the GIF Charité – Universitätsmedizin Berlin and 20 patients of the social-psychiatric outpatient clinic adolescents who met the inclusion criteria of age > = 11 years and completed the BIS were included (*n*_1__TG = 99). Their age ranged between 11 and 18 years (*M* = 15.36, *SD* = 1.85) of whom 25 persons (25.3%) reported to be assigned male at birth (AMAB) and 74 (74.7%) assigned female at birth (AFAB). 68 (68.7%) persons reported to identify as boys, 22 (22.2%) as girls and 9 (9.1%) as non-binary. 86.5% came to the outpatient clinics primarily in search of gender affirming treatment and 13.5% to ask for counseling. At the time of assessment, 68.9% were socially transitioned whilst 31.1% hadn’t had their official coming out yet except among their families. 90.9% reported German as the family language, 9.1% stated other languages such as English, Polish and Russian. All participants were white.

For the analyses among the transgender adolescents only, we had *n*_2__TG = 74 (age *M* = 15.873, *SD* = 1.66), with 17 (23.0%) AMAB and 57 (77.0%) AFAB adolescents. *N* = 53 (71.6.%) adolescents indicated to identify as boys, 15 (20.3%) as girls and 6 (8.1%) as non-binary. For both samples, the gender identity distribution according to each ASAB can be retrieved from Table 1.

**Table 1:** Distribution of assigned sex at birth and gender identity in the two samples used for the analyses

	n_1__TG *N* = 99		n_2__TG *N* = 74	
	AMAB^1^ 25 (25.3%)	AFAB^2^ 74 (74.4%)	AMAB 17 (23.0%)	AFAB 57 (77.0%)
trans girls	22 (22.2%)		15 (20.3%)	
trans boys		68 (68.7%)		53 (71.6%)
non-binary	3 (3.03%)	6 (6.06%)	2 (2.70%)	4 (5.41%)

*Notes. *^1^AMAB = assigned male at birth; ^2^AFAB = assigned female at birth

### Measures

All measures were used at the first entrance of the GIF or outpatient clinic.

#### Body dissatisfaction

To assess body dissatisfaction, we used the German version of the validated Body Image Scale (BIS) according to Pauly and Lindgren [27]. The scale was developed for transgender people to evaluate body image and (dis)-satisfaction with different body regions during treatment requests and after transition steps. By means of a silhouette, 30 body parts are illustrated, each of which is to be rated on a five-point scale regarding one’s subjective satisfaction (1 = very satisfied, 2 = satisfied, 3 = neutral, 4 = dissatisfied, and 5 = very dissatisfied). The BIS is available in two versions, one for the female and one for the male ASAB. It is possible to form a global mean score (BIS-30) for body image across all items, and to form mean scale scores for the superordinate scales primary and secondary sex characteristics (BIS-sex), and non-hormone reactive characteristics (BIS-non-hormonal) [31]. Additionally, the BIS allows the construction of six subscales: social and hair growth, head and neck, muscularity and posture, hip region, breast, and genitals. A summary of the body regions included in the BIS superordinate scales and the subscales is presented in Table 2. The internal consistency for all scales in both samples was in a satisfactory range between $\alpha$=0.91. to 93.

**Table 2:** Overview of BIS scales with descriptions and included body regions

Scale	Description	ASAB^1^	Items
BIS-total	Total score	f/m	all body parts
BIS-sex	Primary sex characteristics	f^2^	vagina, clitoris, ovaries, chest, breasts, facial hair, voice
		m^3^	penis, scrotum, testicles, facial and body hair, chest
	Secondary sex characteristics	f	hips, figure, waist, arms, buttocks, thighs, biceps, stature, weight, overall appearance, body hair, head hair, muscles
		m	hip, figure, waist, arms, buttocks, biceps, overall appearance, stature, musculature, thighs, weight, head hair, voice, chest
BIS-non- hormonal	Hormonally non-reactive characteristics	f/m	nose, shoulders, chin, calves, hands, feet, adam’s apple, eyebrows, face, body size
BIS-social	Social & hair	f/m	Overall appearance, body hair, stature, facial hair, head hair, voice
BIS-head	Head & neck	f/m	Adam’s apple, chin, eyebrows, face, nose
BIS-muscles	Muscularity & posture	f/m	Arms, feet, hands, height, calves, musculature, shoulders, biceps, weight
BIS-hip	Hip region	f/m	Buttock, figure, hips, thighs, waist
BIS-chest	Chest region	f/m	Chest, breasts
BIS-genitals	Genitals		Penis/vulva, testicles/clitoris, scrotum/ovaries

*Notes.*^1^assigned sex at birth; ^2^female; ^3^male.

#### Gender dysphoria

To provide a comprehensive measure of gender dysphoria in social context (SGD) beyond body dissatisfaction, we used the Utrecht Gender Dysphoria Scale (UGDS) [28]. The scale consists of 12 items and has two versions dependent on the ASAB (female-to-male, FtM; male-to-female, MtF). In both versions, nine items cover dysphoria in relation to gender identity (e.g. *I hate myself because I’m a boy; MtF*), to gender roles (*I feel unhappy because I have to behave like a girl, FtM)* and to other people’s misidentification of one’s gender (*I feel unhappy when someone calls me a boy, MtF*) [30]. Three items focus on dissatisfaction with bodily aspects (e.g., *I hate heaving breasts, FtM*). Therefore, the total score on gender dysphoria as assessed with the UGDS covers social context variables to a large extent, and, to a lesser extent, aspects of body dissatisfaction as well. Items are answered on a 5-point scale from agree completely to disagree completely, resulting in a sum score from 12 (minimal dysphoria) to 60 (maximal dysphoria). In a validation study (Steensma et al., 2013) the Cronbach’s alpha of the total score was 0.98 for both versions, in the current sample it was $\alpha$=0.92.

#### Experiences of minority stress

**Peer-relations**. The degree of problems in social interactions with peers is measured using the Poor-Peer-Relations Scale (PPR-Scale) from the Youth Self-Report/11-18R (YSR-R) [27]. The self-report mental health questionnaire covers 99 items on behavioral problems, emotional problems, and somatic complaints on a 3-point scale (not applicable, somewhat or sometimes applicable, exactly or often applicable). The PPR-Scale consist of the three YSR-R items: Item 25 (“I don’t get along with other children”), Item 38 (“I get teased a lot”) and Item 48 (“I am not liked by other children”). The PPR-Scale has already been used in various studies among transgender adolescents [14, 17, 20]. In the present sample, the internal consistency of the scale is Cronbach‘s α = 0.91. A higher PPR score indicates a higher degree of peer problems (range 0–6).

**Trans hostile experiences.** In the first standardized clinical interview at both outpatient services, every adolescent is asked whether they experienced trans hostility in the present or past (“Did you experience trans hostile experiences in the past, e.g., name calling, insults, being misgendered on purpose, and humiliations in classroom students and/or teachers?”). The answers were open ended and coded dichotomously (0 = no, 1 = yes).

### Data analysis

Data were analyzed using SPSS 29. For the first study aim, comparisons of BIS-scores between TG and CG were conducted by univariate analysis of covariance (ANCOVA). Since the age range differed between both groups, age was introduced as a covariate to control its effects, with η^2^ as effect size (η^2^ < 0.06: small effect, η^2^ < 0.14: moderate effect, η^2^ > 0.14 large effect). Additionally, in each group, differences of body dissatisfaction between gender identities were calculated by ANCOVAs under the control of age, too.

Secondly, we explored body dissatisfaction, gender dysphoria and minority stress among a subgroup of the TG sample in more detail. After a descriptive analysis of the variables, associations between body dissatisfaction (i.e., BIS total score and BIS subscales), gender dysphoria (i.e., UGDS) and experiences of minority stress (i.e., poor peer-relation scale) were examined by Spearman’scorrelations. Further, we compared adolescents with and without experiences of trans hostility regarding gender dysphoria, body dissatisfaction and PPR by t-test for independent samples with Cohen’s *d* as effect size (*d* = 0.02 small, *d* = 0.05 moderate, *d* = 0.08 high).

For the third aim, we conducted a multiple, linear regression analysis using first body dissatisfaction and then gender dysphoria as outcome variable, and PPR, gender dysphoria and trans hostile experiences as predictor variables. Age and gender identity were included as covariates to control for possible confounding effects. Based on 1000 bootstrap samples, 95% bias-corrected and accelerated confidence intervals for the coefficient *B* and its standard errors were calculated, which makes the regression robust against violations of homoscedasticity that are often found in small sample sizes as ours. We also tested on lack of fit to check if the relationship between the outcome variable and the predictor variables can be adequately described by the model. For our sample size of *n* = 74 and five predictors, medium effects were assumed (*f*^*2*^ = 0.26) which can be tested with a power of 0.92 (calculated by G*Power).

## Results

### Analysis of body dissatisfaction among the transgender groups and comparison with the school-based group

In line with the hypothesis, transgender adolescents reported significantly higher body dissatisfaction (*p* < .01) across all scales with larger effects (η^*2*^ = 0.07-0.49) than the cisgender control group, i.e., not only in respect to sex characteristics, but to all body parts. The covariate age was significantly related to body dissatisfaction (*p* < .01/0.05) across all scales as well, with small to moderate effect sizes (η^2^ = 0.01-0.02) (Table 3).

**Table 3:** Results of the ANCOVA with body dissatisfaction as dependent variable and age as covariate between control group and TG group

Model	Variable	M_TG_1_^2^ (SD)^3^	M_CG^4^ (SD)	Statistics	Effect Size
1	Age (covariate)	14.43 (0.97)^a^	15.36 (1.85)^b^	F(1,624) = 11.43	*η*^*2*^ = 0.02**
	BIS^1^-sex	65.52 (13.76)	42.17 (12.18)	F(2,624) = 252.19	*η*^*2*^ = 0.29**
2	Age (covariate)	^a^	^b^	F(1,624) = 11.14	*η*^*2*^ = 0.02**
	BIS-non-hormal	39.93 (10.45)	29.58 (9.26)	F(2, 624) = 79.47	*η*^*2*^ = 0.11**
3	Age (covariate)	^a^	^b^	F(1,624) = 10.89	*η*^*2*^ = 0.02**
	BIS-total	96.55 (20.89)	66.08 (18.18)	F(2, 624) = 189.22	*η*^*2*^ = 0.23**
4	Age (covariate)	^a^	^b^	F(1,624) = 10.66	*η*^*2*^ = 0.02**
	BIS-social	19.25 (5.12)	12.8 (3.99)	F(2, 624) = 166.32	*η*^*2*^ = 0.21**
5	Age (covariate)	^a^	^b^	F(1,624) = 6.32	*η*^*2*^ = 0.01*
	BIS-head	13.31 (3.67)	10.53 (3.18)	F(2, 624) = 48.27	*η*^*2*^ = 0.07**
6	Age (covariate)	^a^	^b^	F(1,624) = 13.89	*η*^*2*^ = 0.02**
	BIS-muscles	30.00 (8.04)	22.24 (6.66)	F(2, 624) = 83.0	*η*^*2*^ = 1.12**
7	Age (covariate)	^a^	^b^	F(1,624) = 7.73	*η*^*2*^ = 0.01**
	BIS-hip	16.87 (4.65)	12.42 (4.78)	F(2, 624) = 57.73	*η*^*2*^ = 0.09**
8	Age (covariate)	^a^	^b^	F(1,624) = 4.21	*η*^*2*^ = 0.01*
	BIS-chest	8.26 (1.91)	4.42 (1.72)	F(1, 624) = 352.42	*η*^*2*^ = 0.36**
9	Age (covariate)	^a^	^b^	F(1,624) = 4.50	*η*^*2*^ = 0.01*
	BIS-genitals	12.60 (2.94)	6.31 (2.429	F(2, 610) = 604.65	*η*^*2*^ = 0.49**

*Notes*. ^1^Body Image Scale, ^2^Mean of transgender group, ^3^Standard deviation, ^4^Mean of control group; ^a^covariate age in TG group M = 14.43 (0.97); ^b^ covariate age in CG group M = 15.36 (1.85)

**Fig. 2:**
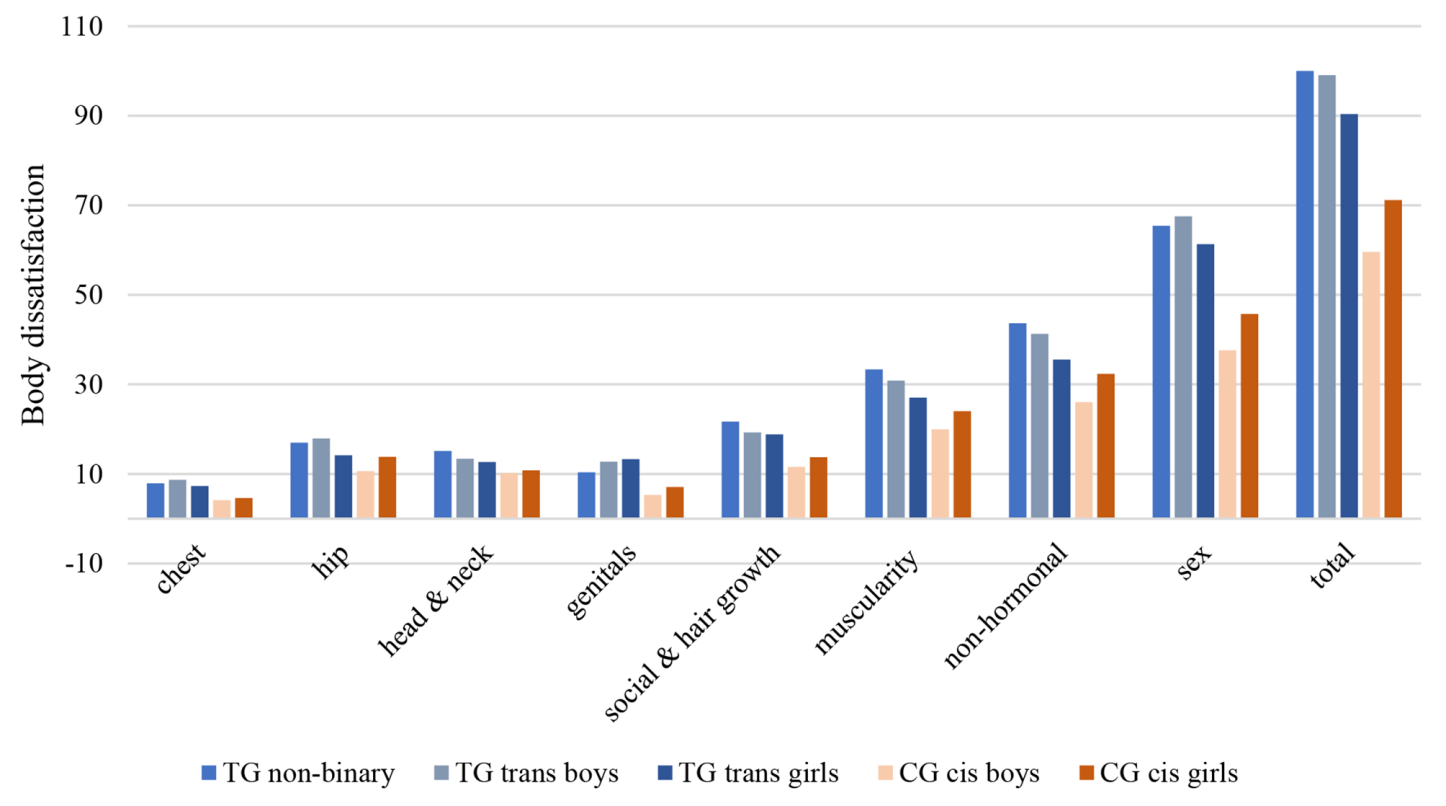
Bar diagram of body dissatisfaction in relation to gender identities for the school-based control group and the clinic-referred transgender adolescents. *Notes.*^1^Body Image Scale. Trans girls, trans boys and non-binary people from clinic-referred transgender (TG) sample. Cisgender boys and cisgender girls from school-based control group (CG)

Figure 2 displays the results for body dissatisfaction in relation to the gender identities in the TG and in the CG. In the transgender group, gender identity differences emerged for the subscales chest, hip region and genitals, with the following pattern: transgender boys reported significantly higher dissatisfaction with their chest (η^*2*^ = 0.08, *p* < .05) and hip region (η^*2*^ = 0.10, *p* < .05) compared to trans girls and non-binary adolescents. Transgender girls stated significantly higher dissatisfaction with their genitals than transgender boys and than non-binary adolescents (η^*2*^ = 0.07, *p* < .05). On the other single subscales, the superordinate scales (sex and nonhormonal body characteristics) or on the total score, no significant differences between gender identities were found (*p* > .05) within the transgender group (Appendix 2). In the control group, in contrast, cisgender girls reported significantly higher body dissatisfaction among all scales (Appendix 3).

### Descriptive data on minority stress, body dissatisfaction and gender dysphoria among the transgender sample

Among the smaller transgender analysis group (transgender_n_*2*_ = 74), the mean score on the BIS was *M* = 97.64 (*SD* = 19.97, min = 46.0, max = 136.0) and the mean score on the UGDS for gender dysphoria was for AFAB *M* = 47.00 (*SD* = 5.39, min = 36.0, max = 76.0) and for AMAB *M* = 47.81 (*SD* = 16.75, min = 21.0.0, max = 93.0). Regarding minority stress, 54.1% had experienced trans hostility in the present and/or past and 63.5% indicated to have PPR at least *somewhat/sometimes* (*M* = 1.41, *SD* = 1.56, min = 0, max = 6).

### Correlations between body dissatisfaction, gender dysphoria and poor peer-relations

The results of Spearman’s correlation (Appendix 4) showed significant but weak correlations between body dissatisfaction and PPR, only for the subscales BIS-social (ρ=0.24, *p* < .05) and BIS-muscles (ρ=0.24, *p* < .05). There were significant moderate correlations between gender dysphoria and BIS-chest (ρ=0.40, *p* < .01), BIS-sex (ρ=0.33, *p* < .01), BIS-total (ρ=0.32, *p* < .01), BIS-non-hormonal (ρ=0.30, *p* < .05), and weak correlations with BIS-genitals (ρ=0.26, *p* > .05) and BIS-hip (ρ=0.25, *p* > .05). There was no significant correlation between gender dysphoria and PPR (*p* > .05). Correlations within the BIS-subscales were high and significant (*p* < .01).

### Mean difference in body dissatisfaction and gender dysphoria regarding experience of trans hostility

Adolescents who had experienced trans hostility reported significantly higher gender dysphoria (*M* = 49.35, *SD* = 9.97) than the group who has not experienced trans hostility (*M* = 44.62, *SD* = 6.93), with a moderate effect size (*d*=-0.54; t(72)=-2.33, *p* < .05; BCa CI-=-8.78, CI+=-0.68). Adolescents with trans hostile experiences reported PPR significantly more often (*M* = 2.00, *SD* = 1.73) than the other group (*M* = 0.74, *SD* = 0.99), with a high effect size (*d* = 1.44 (t(72)=-3.773, *p* < .01; BCa CI-=-1.93, CI+=-0.60). For body dissatisfaction, no significant difference regarding the experience of trans hostility was found (*p* > .05).

### Regression analysis on body dissatisfaction and gender dysphoria

Table 4 provides results for the multiple linear regression on body dissatisfaction as outcome variable with model with age and gender identity as covariates, and gender dysphoria (UGDS total score), PPR-score and trans hostile experiences as predictor variables. The model was able to explain 22.4% of the variance of the outcome variable with a significant F-ratio (*p* < .01) indicating an improvement of prediction by the fitted model. Age and PPR turned out to be significant predictors for body dissatisfaction (*p* < .05).

**Table 4:** Multiple regression model with body dissatisfaction as outcome variable in the transgender group (*n* = 74)

	B^3^ (SE)^4^	β^5^	CI-, C + ^6^
(constant)	13.11 (23.15)		[-33.09, 59.30]
**Age**	**4.01 (1.31)**	0.33*	**[1.40, 6.63]**
Gender	-3.15 (2.66)	-0.14	[-8.46, 2.16]
PPR^1^	3.18 (1.62)	0.25	[-0.05, 6.41]
Trans hostility	-3.25 (4.92)	-0.08	[-13.08, 6.57]
UGDS^2^	0.41 (0.25)	0.19	[-0.09, 0.92]

*Notes.* ^1^Poor-Peer-Relations; ^2^Utrecht Gender Dysphoria Scale; ^4^bias corrected and accelerated confidence interval of B; Confidence intervals and standard errors based on 1000 bootstrap samples. Model 1: adjusted *R*^2^ = 0.13, F(2) = 6.41, *p* < .05*; Model 2: adjusted *R*^2^ = 0.17, F(5) = 3.9, *p* < .05*

Table 5 demonstrates results for multiple linear regression on gender dysphoria. Again, age and gender as covariates and body dissatisfaction, PPR and trans hostility as predictors were added to the model. Trans hostility was the only significant predictor for gender dysphoria (*p* < .05). The model was able to explain 15.1% of the variance of the outcome variable with a significant F-ratio also indicating an improvement of prediction by the fitted model (*p* < .05).

**Table 5:** Multiple regression model with gender dysphoria as outcome variable in the transgender group (*n* = 74)

	B (SE)	b	BCa 95% CI-, CI + ^4^
(constant)	38.21 (17.22)		[10.18, 77.30,]
Age	-0.08 (0.70)	-0.01	[-1.77, 1.40]
GID^1^	-1.23 (1.28)	-0.12	[-3.36, 1.52]
PPR^2^	-1.01 (0.61)	-0.18	[-2.38, 0.14]
**Trans hostility**	**6.13 (2.22)**	0.34*	**[2.29, 10.46]**
BIS-total^3^	0.09 (0.08)	0.20	[-0.09, 0.21]

*Notes. *^1^Gender identity^; 2^Poor-Peer-Relations; ^3^Utrecht Gender Dysphoria Scale; ^4^bias corrected and accelerated confidence interval of B; Confidence intervals and standard errors based on 1000 bootstrap samples. *R*^2^ = 0.151, F(5) = 2.43, *p* < .05*

## Discussion

The aim of the current paper was to gain a better understanding of body dissatisfaction and gender dysphoria in the social context of minority stress among clinic-referred transgender adolescents. We first compared body dissatisfaction in our sample with data from a nonclinical cisgender-gender control group and hereby explored the dissatisfaction with specific body regions and gender identity effects. Second, we analyzed associations between body dissatisfaction, gender dysphoria and experiences of minority stress, defined as poor peer-relations and trans hostile experiences on the bivariate and multivariate level.

The first analyses showed that transgender adolescents reported significantly higher body dissatisfaction on all BIS scales compared to cisgender adolescents. That finding is in line with a matched-control study among adults that found higher body dissatisfaction among transgender individuals than cisgender adults [9]. Highest effect sizes were found for muscles and genitals. A vast amount of studies shows that the incongruence between body and gender identity causes great dissatisfaction (for instance [31]). Additionally, some studies have pointed out that especially body parts that are salient when socially negotiating gender identity and being categorized by others, are causing dissatisfaction [32]. Our results add to this notion as they show that it is not only the body parts related to the sex characteristics that cause dissatisfaction but also the body parts and aspects of appearance that are merely visible to others.

Regarding gender identity differences, results for the control group were in line with the literature, as cisgender girls reported being more unsatisfied with their body than cisgender boys [33]. Within the transgender group however, a specific pattern of gender identity differences emerged: trans girls showed higher dissatisfaction with their genitals and trans boys with their hip and chest region compared to the respective other gender identities. These findings are somewhat reasonable as wide hips and breasts are highly associated with femininity, in turn, penis and scrotum with masculinity [8, 34, 35]. Previous research on differences in body dissatisfaction in relation to gender identity among transgender samples are inconsistent as some studies report higher total scores of dissatisfaction for trans boys [9, 10], others for trans girls [6]. Some studies reported less dissatisfaction for non-binary people [36] whilst in our sample there is a tendency of higher dissatisfaction for non-binary adolescents. These different results can be attributed to various factors such as timing and status of social and medical transition, reality of life [10], social environment, extent of passing [37, 38] or location and year of survey [39]. Accordingly, caution should be exercised in making generalized statements and more research is needed to gain more knowledge on correlates of gender differences in body dissatisfaction among transgender adolescents.

To further understand the experiences of body dissatisfaction and its correlates, further analyses were conducted with the transgender subsample. There were correlations between gender dysphoria and the total score for body dissatisfaction, the superordinate scores sex characteristics and hormonally non-reactive body characteristics and the subscale scores chest, genitals and hips. While comparably detailed analyses are lacking in the literature, results from another study correspond to our finding, as they showed that the majority of trans boys suffer from the presence of their breasts, which is why many of them wear a binder for compression and wish to get a mastectomy [40].

Our results also show that the association between body dissatisfaction and gender dysphoria did not apply to the body parts captured by the subscale muscles (arms, feet, hands, height, calves, musculature, shoulders, biceps, weight) and the social subscale (overall appearance, body hair, stature, facial hair, head hair, voice). Interestingly, we found positive but low correlations between poor peer relations and body dissatisfaction for these two scales, BIS-social scale (ρ=0.24, *p* < .05) and the BIS-muscles scale (ρ=0.24, *p* < .05). This finding is supported by studies showing that body features which are quickly visible in everyday interactions, and especially those that are not associated with one’s gender identity, are strongly negatively evaluated [8, 9]. The perception, evaluation and presentation of one’s body parts is related to the aspect of visual conformity with the affirmed gender, defined as passing [38, 41]. Accordingly, one’s body characteristics are directly related to one’s visibility as a trans person or to pass as cisgender person in public. The specific relationship between passing and body dissatisfaction has not been examined yet in the literature.

The finding that poor peer relations correlate with the subscales muscle and social for body dissatisfaction are in line with results from other studies on the relationship between body dissatisfaction and experiences of being harassed, excluded or socially isolated [2, 23] and experiences of social distress and minority stress [23, 42, 43] among transgender samples. In turn, social acceptance was found to be a protective factor for body satisfaction and was closely related to self-acceptance and self-compassion among transgender adolescents [23, 42, 43]. This underlines the need to consider resilience-oriented, protective factors in future analyses in the context of minority stress among transgender persons to gain more knowledge on target points for interventions addressing body dissatisfaction.

In contrast and against hypothesis, poor peer relations and gender dysphoria were not correlated in our sample (*p* > .05). A hypothesis is that general, gender- unspecific social distress like poor peer relations might not directly affect gender dysphoria and might rather affect the evaluation of one’s (visible) body, which might often be the target of the kind of bullying captured by the poor peer relation scale. Corresponding to this notion, the relation between general bullying and body dissatisfaction was also found among cisgender adolescents [44]. Due to the lack of comparable analyses, replication of and reasons for this pattern remain subject to future research.

Regarding trans hostility, results point to a reversed pattern and hypotheses were partly confirmed as well: While there was no difference in the level of body dissatisfaction, the group of trans adolescents who had experienced trans hostility experienced significantly higher gender dysphoria than the group without trans hostile experiences, with medium effect size (*d* = 0.57). This finding indicates that trans specific social minority stress specifically interacts with the dysphoria related to gender role and gender identity, which has already been confirmed in adult samples [2, 32], and less with body dissatisfaction. As stated in the *minority stress model* [13, 19] and shown in other large-scale studies on hostility against members of the LGBTIQ (lesbian, gay, bisexual, transgender, inter, queer) community [45], discrimination, microaggressions, hatred and violence based on a group affiliation (be it gender, race, sexuality, etc.) can have serious consequences for a person’s mental health and well-being, as one’s identity is specifically attacked. Adding to our finding on the relation to gender dysphoria, trans hostile experiences present as an important target point in the context of counseling and psychosocial support for transgender persons.

In the fourth analysis, we aimed to identify correlates of body dissatisfaction and gender dysphoria on a multivariate level. In the regression on body dissatisfaction, we found that the older the adolescents, the more dissatisfied they were with their body. The important role of age regarding body dissatisfaction can be explained by the start of puberty and the body’s development in an undesired direction, which is a painful and stressful experience for many transgender adolescents [46, 47]. Therefore, puberty often marks a starting point of body-specific gender dysphoria for e.g., when trans boys have their first period or trans girls enter the voice change [12]. Moreover, PPR was found as a predictor for total body dissatisfaction whilst gender dysphoria and trans hostile experiences were not. This finding extends the correlation analyses as it shows that poor peer relations are specifically related to body dissatisfaction, beyond trans hostility and gender dysphoria.

Following on from this, in the regression on gender dysphoria, trans hostility was the only significant predictor in the regression analyses (*p* < .05). Taking together, the bivariate correlations and the multivariate analyses present a specific pattern: both body dissatisfaction and gender dysphoria are related to aspects of minority stress, however, trans-hostility, i.e., gender specific hostility, seems to be more strongly connected to gender role dysphoria while more general problems with peers are more connected with body dissatisfaction.

The patterns found in this study cautiously suggest two different but complementing experiences of dysphoria among transgender youth. In line with this notion, two recent large-scale community-based studies among transgender adults considered gender dysphoria and body dissatisfaction in the context of a (trans hostile) societal environment. Results point to the differential perception of body-specific gender dysphoria **(**BGD**)** and gender dysphoria in social context **(**SGD**)** [4, 32, 48]: Based on qualitative data from 463 trans and non-binary adults aged 18–74 years, Pulice-Farrow et al. [32] defined BGD as feelings of disconnection from the body and manifestations of distress in relation to one’s body. BGD, however, may also vary in different social contexts. For example, “body dysphoria was often described as being triggered by situational and gendered expectations, and became salient when having to socially negotiate identity” [32]. Another qualitative study of 610 trans adults showed that gender dysphoria can be highly impacted by social context and that external factors such as gender-based rejection and discrimination may elicit feelings of gender dysphoria that were absent before. For example, a trans woman reported that she felt good about her voice and loved singing until she was frequently misgendered because of its timbre [4]. While the difference between BGD and SGD might not always be easy to disentangle [32], this is an important new approach to stimulate research on the complex dysphoric experiences of both gender dysphoria and body dissatisfaction [4, 32].

### Limitations

One limitation of this study was that we had to work with two samples due to incomplete questionnaire sets. Both had a small sample size, especially of the second subsample of transgender youth (TG_*n*_2_ = 74). Furthermore, it should be considered that the transgender sample was already affiliated to a specialized gender counseling and therefore this sample cannot be representative for all trans adolescents in Germany, e.g., as the level of parent’s support may have been higher in our treatment seeking sample and youth with less supportive parents might suffer from worse mental health [49]. Also, there was a large disbalance between gender identities in the sample, with a majority of trans boys (71.6%). This finding is somewhat congruent with the current literature showing that more trans boys than trans girls or non-binary individuals are consulting special services in Europe [14, 20, 50]. It is explained by the assumption that trans girls have their coming-out later because it is more difficult and less socially accepted [51]. However, since some studies show that especially trans girls [19, 52] as well as non-binary and gender-queer individuals [45] suffer from discrimination and violence it is important that their perspectives are represented in future research. This sample was not diverse either, since the majority was white and with a German background. Using an intersectional^1^ lens, especially when investigating body dissatisfaction, is crucial when it comes to societal discriminatory effect powers. Since bodies can be understood as places of power dynamics’ action and one’s relation to a body is affected by social meanings and practices [54] for e.g. Black, Indigenous people, trans people with disabilities or poor trans individuals should be taken into account [55]. Additionally, the control group was assessed in 2006–2007 and more recent, representative assessments of both gender identity and body dissatisfaction among cisgender and transgender adolescents are needed to exclude time effects.

Regarding our used instruments, the UGDS has been criticized by the trans community for its two binary and different versions for AMAB/AFAB and because gender dysphoria is not reflected in its complexity. Therefore, literature points out that the unrevised version of UGDS as used in this study was found to have measurement errors especially for non-binary people and that some trans persons do not feel represented well by the questionnaire’s constructs [56, 57]. In future research, the revised version UGDS-GS should be used [56] but more adequate instruments to assess gender dysphoria in social contexts for adolescence are needed as well [58]. Moreover, our operationalization of minority stress experiences was constructed using the available measures for peer relations and trans hostile experience, leaving other aspects of proximal or distal minority stress like discrimination and non-affirmation behind [59]. Specific instruments such as the validated Gender Minority Stress and Resilience Measure [60] might offer more elaborated ways of assessing minority stress and should be considered for future research. However, instruments are mostly constructed for trans adults and adequate instruments for trans youth are lacking [57].

With respect to our results, it should be noted that our regression models for both outcome variables explained little variance, 22.4% for body dissatisfaction and 15.1% gender dysphoria. We can only speculate about reasons for this low explained variance in the outcomes. Besides the restricted sample size, the quality of measures of our predictors might have been insufficient, or the models neglected important predictors of gender dysphoria and body dissatisfaction. Regarding recent changes in terminology and variation in gender identifications there is a need for more up-to-date instruments that capture transgender adolescent’s experiences adequately which will then inform future research and allow new hypotheses and conceptualizations.

### Implications and prospect

The current paper gives novel indications: first, body dissatisfaction with respect to all areas of the body and outer appearance among transgender adolescents aged 11–18 years is higher than among cisgender adolescents in a comparable age range 13–16 years. Further, gender differences are far less consistent in the transgender group than in the cisgender group pointing to specific developments and experiences. Second, as among transgender adolescents, body dissatisfaction and gender dysphoria are associated with experiences of minority stress, these experiences cannot be explored without taking social interactions and societal positions, i.e., being part of a minority, marginalized and stigmatized group, into consideration. In this respect, when addressing gender dysphoria in clinical practice and in future research, social influences and experiences of trans hostility as deeply invalidating stressors need to be taken into account. This is of high relevance as a recent study found that internalized transphobia predicts worse outcomes regarding body uneasiness after GAHT, which is the standard of care for transgender adolescents with gender dysphoria and body dissatisfaction in particular cases [61, 62]. Additionally, in the context of body dissatisfaction and gender dysphoria, it would be important to explore the role of passing, i.e., their perception and satisfaction of congruence between outward appearance and gender identity. A few studies have attempted to measure passing, for which they have constructed their own scales [38, 41]. However, the role of passing for gender dysphoria has hardly been examined, conceivably since validated measurements for the assessment of passing are only emerging. There is, for instance, the Transgender Identity Survey measuring internalized transphobia with a subscale that assesses passing [41]. To our knowledge, validated instruments for adolescents are lacking so far.

To gain more knowledge of trans adolescents’ lived experiences, a participatory research approach is needed [63]. Dismantling gate-keeping positions by considering transgender clients as experts for their own experience is just one of many steps that still must be taken in order to destigmatize and de-pathologize gender and sexual diversity. Further, longitudinal studies are needed to identify risk and protective factors of gender dysphoria and body dissatisfaction in relation to the social context and minority stress.

## Conclusions

This study indicates that clinic-referred transgender adolescents show higher body dissatisfaction for all body regions than their cisgender peers. Experiences of minority stress such as poor peer relations and trans hostility are specifically associated with body dissatisfaction and with gender dysphoria. Adequate, validated and revised instruments - in line with recent changes in terminology and classification - to assess body dissatisfaction, gender dysphoria and aspects of minority stress are needed, especially for the age group of adolescents. While this research needs time, clinicians need to be aware of the societal impact on gender minority individuals, address this in the counseling setting and provide support for the youth.

### Electronic supplementary material

Below is the link to the electronic supplementary material.

Supplementary Material 1

## Data Availability

The data presented in this study are available on appropriate request from the corresponding author. The data are not publicly available as the privacy of the human subjects must be ensured.

## Footnotes

1 Intersectionality means the intersection between different forms of discrimination such as racism, sexism, ablesim and classism that interact with each other and lead to a conglomerate of adverse social and health outcomes [53].

## Abbreviations

ASAB: assigned sex at birth
AFAB: assigned female at birth
AMAB: assigned male at birth
BIS: Body Image Scale
BGD: body–specific gender dysphoria
CG: school–based control group
PPR: poor peer relations
SGD: gender dysphoria in social context
TG: transgender group
transgender: transgender, non–binary, genderqueer
UGDS: Utrecht Gender Dysphoria Scale
YSR-R: Youth–Self–Report
