# Supplementary material for: Analyzing body dissatisfaction and gender dysphoria in the context of minority stress among transgender adolescents
Source: Child Adolesc Psychiatry Ment Health. 2024 Mar 2;18:30. doi: 10.1186/s13034-024-00718-y (PMC10909265; doi:10.1186/s13034-024-00718-y)
Supplement: Supplementary file 1 — Supplementary Material 1 [file 13034_2024_718_MOESM1_ESM.docx]

**Appendix**

**Manuscript**

**Analyzing body dissatisfaction and gender dysphoria in the context of minority stress among transgender adolescents**

**Authors**

Alexandra Brecht^1,2^*, Sascha Bos^2^, Laura Ries^2^, Kerstin Hübner^2^, Pia-Marie Widenka^3^, Sibylle Maria Winter**^2^ and Claudia Calvano**^1^

^1^ Department of Education and Psychology, Clinical Child and Adolescent Psychology and Psychotherapy, Freie Universität Berlin, 14195 Berlin, Germany

^2^ Department of Child and Adolescent Psychiatry, Charité—Universitätsmedizin Berlin, corporate member of Freie Universität Berlin, Humboldt Universität zu Berlin and Berlin Insitute of Health, 13353 Berlin,

Germany

^3^ Universität zu Lübeck, Klinik für Kinder- und Jugendmedizin, Ratzeburger Allee 160, 23562 Lübeck, Germany

*Correspondence: [alexandra.brecht@charite.de](mailto:alexandra.brecht@charite.de)

**Shared senior authorship

**Appendix 1**

**Descriptives on school-based control group – data of the Intersexuality Network**

The control data for this paper were derived from the evaluation study in the Disorder of Sex Development/Intersexuality Network, funded by the German Federal Ministry of Education and Research [29]. This study investigated the effects of variations of sex development, the associated assessments, and diagnoses as well as medical and surgical interventions on physical, psychological, and social health. As part of the project, a control group was recruited to record the situation of adolescents without a variation of sex development. The control group consisted of secondary school and grammar school students in grades 7 to 10 and was recruited from January 2006 to March 2007 in the German federal states Bremen and Schleswig-Holstein, both from rural and urban areas. Prior approval was obtained from relevant Ministries (i.e., the Senator for Education and Science in Bremen, the Ministry for Education, Science, Research and Culture in Schleswig-Holstein, the state commissioner for Data Protection and Freedom of Information of the Free Hanseatic City of Bremen and the Independent State Center for Data Protection Schleswig-Holstein). Regarding informed consent and data collection, in Bremen, the permission of the school principal, the parents' and pupils' councils as well as the signed information forms and consent forms of the parents and young people had to be obtained. In Schleswig-Holstein, the data was processed anonymously according to the specifications of the independent Schleswig-Holstein State Center for Data Protection without a signed consent form, but with the consent of young people and parents to be marked with a cross. From the control group data set, the BIS data from the population-based control group will be used in the present work. Sociodemographic data are used for sample description. We included cases ranging between 13-16 years of age for the highest age match with the clinical GIF sample.

**Appendix 2.** Results of the ANCOVA with body dissatisfaction as independent variable and age as covariate between gender identities within the TNG group

| **Model** | **Variable** | ***M*_boys^2^ *(SD)^3^*** | ***M*_girls^4^ (*SD*)** | **M_non-binary (SD)** | **Statistics** | **Effect Size** |
| --- | --- | --- | --- | --- | --- | --- |
| 1 | Age (covariate) | 15.39 (1.78)^a^ | 15.09 (2.17)^b^ | 15.81 (1.60) | F(1, 98) = 21.73 | *η^2^* = .21** |
|  | BIS^1^-sex | 66.88 (14.09) | 61.32 (13.50) | 65.44 (10.68) | F(2, 98) = 1.22 | *η^2^* = .03 |
| 2 | Age (covariate) | ^a^ | ^b^ |  | F(1,98) = 16.39 | *η^2^* = .15** |
|  | BIS-non-hormal | 40.85 (10.93) | 35.55 (9.31) | 43.67 (5.75) | F(2, 98) = 2.41 | *η^2^* = .05 |
| 3 | Age (covariate) | ^a^ | ^b^ |  | F(1,98) = 21.49 | *η^2^* = .18** |
|  | BIS-total | 98.09 (21.65) | 90.36 (20.83) | 100.00 (11.83) | F(2, 98) = .94 | *η^2^* = .02 |
| 4 | Age (covariate) | ^a^ | ^b^ |  | F(1, 98) = 18.58 | *η^2^* = .16** |
|  | BIS-social | 19.07 (5.21) | 18.82 (5.47) | 21.67 (2.78) | F(2, 98) = 0.82 | *η^2^*= .02 |
| 5 | Age (covariate) | ^a^ | ^b^ |  | F(1,98) = 16.74 | *η^2^* = .15** |
|  | BIS-head | 13.28 (3.78) | 12.68 (3.66) | 15.11 (2.26) | F(2, 624) = 1.01 | *η^2^* = .02 |
| 6 | Age (covariate) | ^a^ | ^b^ |  | F(1, 98) = 13.77 | *η^2^* = .13** |
|  | BIS-muscles | 30.51 (8.39) | 27.05 (7.51) | 33.33 (3.87) | F(2, 98) = 1.98 | *η^2^* = .04 |
| 7 | Age (covariate) | ^a^ | ^b^ |  | F(1, 98) = 15.13 | *η^2^* = .14** |
|  | BIS-hip | 17.72 (4.77) | 14.18 (3.78) | 17.00 (3.35) | F(2, 98) = 5.13 | *η^2^* = .10* |
| 8 | Age (covariate) | ^a^ | ^b^ |  | F(1, 98) = 11.88 | *η^2^* = .11** |
|  | BIS-chest | 8.26 (1.91) | 4.42 (1.72) | 7.89 (1.90) | F(1, 98) = 4.32 | *η^2^* = .08* |
| 9 | Age (covariate) | ^a^ | ^b^ |  | F(1,97) = 6.29 | *η^2^* = .06* |
|  | BIS-genitals | 12.63 (2.93) | 13.32 (2.50) | 10.37 (3.38) | F(2, 97) = 3.71 | *η^2^* = .07* |

^1^Body Image Scale, ^2^Mean of TNG group, ^3^Standard deviation, ^4^Mean of control group

**Appendix 3.** Results of the ANCOVA with body dissatisfaction as independent variable and age as covariate between gender identities within the control group

| **Model** | **Variable** | ***M*_boys^2^ *(SD)^3^*** | ***M*_girls^4^ (*SD*)** | **Statistics** | **Effect Size** |
| --- | --- | --- | --- | --- | --- |
| 1 | Age (covariate) | 14.45 (.98)^a^ | 14.43 (.97)^b^ | F(1, 526) = 1.41 | *η^2^* = .003 |
|  | BIS^1^-sex | 37.63 (12.10) | 45.72 (11.02) | F(2, 526) = 64.38 | *η^2^* = .11** |
| 2 | Age (covariate) | ^a^ | ^b^ | F(1, 526) = 2.11 | *η^2^* = .004 |
|  | BIS-non-hormal | 26.03 (8.68) | 32.35 (8.76) | F(2, 526) = 68.49 | *η^2^* = .12** |
| 3 | Age (covariate) | ^a^ | ^b^ | F(1, 526) = .97 | *η^2^* = .002 |
|  | BIS-total | 59.58 (18.35) | 71.16 (16.37) | F(2, 526) = 58.43 | *η^2^* = .10** |
| 4 | Age (covariate) | ^a^ | ^b^ | F(1, 526) = .60 | *η^2^* = .001 |
|  | BIS-social | 11.59 (3.98) | 13.75 (3.73) | F(2, 526) = 40.89 | *η^2^*= .07** |
| 5 | Age (covariate) | ^a^ | ^b^ | F(1, 526) = .01 | *η^2^* = .00 |
|  | BIS-head | 10.18 (3.31) | 10.80 (3.06) | F(2, 526) = 4.98 | *η^2^* = .01* |
| 6 | Age (covariate) | ^a^ | ^b^ | F(1, 526) = 4.16 | *η^2^* = .01* |
|  | BIS-muscles | 19.97 (6.55) | 24.02 (6.19) | F(2, 526) = 53.16 | *η^2^* = .09** |
| 7 | Age (covariate) | ^a^ | ^b^ | F(1, 526) = 1.44 | *η^2^* = .003 |
|  | BIS-hip | 10.64 (4.16) | 13.81 (4.77) | F(2, 526) = 64.17 | *η^2^* = .11** |
| 8 | Age (covariate) | ^a^ | ^b^ | F(1, 526) = .03 | *η^2^* = .00 |
|  | BIS-chest | 4.17 (1.80) | 4.62 (1.63) | F(2, 526) = 8.98 | *η^2^* = .02* |
| 9 | Age (covariate) | ^a^ | ^b^ | F(1, 526) = 1.39 | *η^2^* = .003 |
|  | BIS-genitals | 5.33 (2.23) | 7.10 (2.28) | F(2, 526) = 78.43 | *η^2^* = .13** |

^1^Body Image Scale, ^2^Mean of TNG group, ^3^Standard deviation, ^4^Mean of control group

**Appendix 4.** Spearman’s correlations between body dissatisfaction and gender dysphoria among transgender group (*n*=74)

|  | BIS-total | BIS-sex | BIS-non-hormonal | BIS-social | BIS-head | BIS-muscles | BIS-hip | BIS-genitals | BIS-chest | UGDS^3^ |
| --- | --- | --- | --- | --- | --- | --- | --- | --- | --- | --- |
| PPR^1^ | 0.20 | 0.18 | 0.19 | **.24^*^** | 0.12 | **.24^*^** | 0.07 | 0.17 | -0.03 | -0.06 |
| BIS^2^-total | 1.00 | **.96^**^** | **.95^**^** | **.88^**^** | **.70^**^** | **.88^**^** | **.79^**^** | **.45^**^** | **.60^**^** | **.32^**^** |
| BIS-sex |  | 1.00 | **.94^**^** | **.86^**^** | **.56^**^** | **.85^**^** | **.80^**^** | **.52^**^** | **.66^**^** | **.35^**^** |
| BIS-non-hormonal |  |  | 1.00 | **.82^**^** | **.61^**^** | **.91^**^** | **.84^**^** | **.35^**^** | **.53^**^** | **.30^*^** |
| BIS-social |  |  |  | 1.00 | **.62^**^** | **.75^**^** | **.56^**^** | **.34^**^** | **.50^**^** | **.33^**^** |
| BIS-head |  |  |  |  | 1.00 | **.53^**^** | **.52^**^** | 0.14 | **.35^**^** | 0.17 |
| BIS-muscles |  |  |  |  |  | 1.00 | **.68^**^** | **.27^*^** | **.45^**^** | 0.21 |
| BIS-hip |  |  |  |  |  |  | 1.00 | **.32^**^** | **.43^**^** | **.25^*^** |
| BIS-genitals |  |  |  |  |  |  |  | 1.00 | **.37^**^** | **.26^*^** |
| BIS-chest |  |  |  |  |  |  |  |  | 1.00 | **.40^**^** |
| UGDS^3^ |  |  |  |  |  |  |  |  |  | 1.00 |

*Notes.* ^1^Poor-Peer-Relation-Scale; ^2^Body-Image-Scale; ^3^Utrecht Gender Dysphoria Scale; **p*<.05; ***p*<.01.
